# Impact of structure space continuity on protein fold classification

**DOI:** 10.1038/srep23263

**Published:** 2016-03-23

**Authors:** Jinrui Xu, Jianzhi Zhang

**Affiliations:** 1 Department of Computational Medicine and Bioinformatics, University of Michigan, Ann Arbor, MI 48109, USA; 2 Department of Ecology and Evolutionary Biology, University of Michigan, Ann Arbor, MI 48109, USA

## Abstract

Protein structure classification hierarchically clusters domain structures based on structure and/or sequence similarities and plays important roles in the study of protein structure-function relationship and protein evolution. Among many classifications, SCOP and CATH are widely viewed as the gold standards. Fold classification is of special interest because this is the lowest level of classification that does not depend on protein sequence similarity. The current fold classifications such as those in SCOP and CATH are controversial because they implicitly assume that folds are discrete islands in the structure space, whereas increasing evidence suggests significant similarities among folds and supports a continuous fold space. Although this problem is widely recognized, its impact on fold classification has not been quantitatively evaluated. Here we develop a likelihood method to classify a domain into the existing folds of CATH or SCOP using both query-fold structure similarities and within-fold structure heterogeneities. The new classification differs from the original classification for 3.4–12% of domains, depending on factors such as the structure similarity score and original classification scheme used. Because these factors differ for different biological purposes, our results indicate that the importance of considering structure space continuity in fold classification depends on the specific question asked.

Since the 1970s, classification of protein domain structures has gained wide popularity because of its utility in predicting protein function and studying protein evolution^1^. Many hierarchical classifications of domain structures have been developed^2^,^3^,^4^,^5^,^6^. Among them, SCOP^5^ and CATH^3^,^4^,^7^ databases are commonly regarded as the gold standards because of their substantial manual inspections. The hierarchical levels of SCOP from bottom to top are family, superfamily, fold, and class. In general, families and superfamilies consist of domains that are homologous or structurally very similar. Folds comprise superfamilies of domains with similar secondary structure compositions, orientations, and connection orders. Classes, as the top level, include folds with similar secondary structure compositions. In CATH, the hierarchies are homology superfamily (H), topology (T), architecture (A), and class (C). The H, T, and C levels in CATH are respectively equivalent to the superfamily, fold, and class levels in SCOP. Fold in SCOP or T in CATH is of special interest to structural biologists because members of a fold are structurally similar yet usually have no detectable protein sequence similarity^8^,^9^,^10^,^11^. Thus, fold classification can provide significant insights into protein function and evolution that are beyond the realm of sequence analysis.

The current fold classification in SCOP and CATH implicitly assumes that different folds represent isolated islands in the structure space. This assumption was based on early visual observations from a small number of folds that are structurally highly dissimilar. With the explosion of the number of solved domain structures and the use of structure similarity metrics, increasing evidence supports the concept of a continuous fold space where domains from different folds have significant structural similarities^12^,^13^,^14^,^15^. This discovery prompted multiple authors to question the current fold hierarchy^14^ and propose alternative representations such as structure similarity networks^16^ and maps^17^,^18^. In a network, domains are connected if their structure similarity exceeds an arbitrary threshold, whereas in a map, domains are points in a plane or space reduced from a pairwise structure similarity matrix of all domains. However, none of these new representations are intuitive due to the lack of obvious fold boundaries. As a result, the conventional fold representation still dominates the literature in the study of protein structure-function relationship and protein evolution. Given the wide use of fold classification in many studies^19^, examining the impact of structure continuity on fold classification is important.

Various automatic pipelines have been developed to classify domain structures into folds, and they can be generally divided into two types. The first type directly classifies domains according to their structure and/or sequence similarities with existing folds^20^,^21^,^22^,^23^,^24^,^25^,^26^,^27^. The second type uses a machine learning approach^28^,^29^,^30^,^31^. It first collects positive samples from domain pairs in the same folds and negative samples from randomly paired domains across folds, then trains classifiers on these domain pairs. These classifiers are subsequently used to predict whether a query domain is in the same fold as another domain. To our knowledge, none of the current classification methods explicitly consider fold space continuity. As a result, it is unclear to what degree fold space continuity affects protein structure classification and whether it is legitimate to ignore this continuity in classification.

To answer these questions, we propose and implement a strategy to classify domain structures to existing folds by considering fold space continuity. Briefly, we calculate the likelihood that a structure belongs to a fold by considering the similarity between the structure and the fold as well as the similarities among the structures already classified into the fold. By comparing our new classification with the current CATH and SCOP classifications, we assess the importance of considering the fold space continuity in fold classification.

## Results

### Fold classification without considering within-fold structure heterogeneity

To classify domain structures, we need an objective quantity to measure structure similarities between two domains. TM-score^32^,^33^, calculated by the software TM-align^34^, is chosen for this purpose. High TM-score indicates short average spatial distance between aligned residues in a structure alignment (see Materials and Methods). Unlike many other similarity scores^35^,^36^,^37^,^38^, TM-scores of different domain pairs are directly comparable^32^,^33^,^34^ due to the normalization using either the average sequence length of the two domains under comparison or the length of the shorter domain. The former normalization penalizes the length difference between the two domains, which is appropriate when both domains are complete and comparable (i.e., one is not a subunit of the other). This normalization emphasizes the global structure similarity between domains, and the obtained TM-score is referred to as the global TM-score. By contrast, the latter normalization is appropriate when one domain corresponds to a subunit of the other or when one or both domains are incomplete. We refer to such normalized TM-scores as local TM-scores. Both global and local TM-scores are used in our analyses. After normalization, TM-scores are between 0 and 1. Larger TM-scores indicate higher structural similarities.

We focus primarily on the CATH database in this study because it is updated regularly and contains more recently solved domain structures than other databases. We refer to the T level in CATH as fold, because it is equivalent to the fold level in SCOP. We collected from CATH (version 3.5.0) 21,309 representative domains whose mutual sequence identities are ≤60% and sequence lengths are ≥40 residues. These domains are from 1,158 folds in the CATH classification. Of these folds, 141 comprise at least 25 representative domains each. We used these large folds in subsequent analysis, because smaller folds provide insufficient information for statistical analysis. In spite of the relatively low fraction of folds analyzed here, for two reasons, these large folds are highly likely to cover most continuous regions of the fold space. First, these large folds include 17,043 or 82% of all representative domains. Second, the large folds are closer to one another than they are to the 1017 small folds (*P* < 1.5e−18; Wilcoxon signed-rank test), where the closeness between two folds is measured by the highest TM-score of all domain pairs across the two folds.

We randomly choose 10% of domains from each of the large folds as our query domains, whereas the rest of the domains stay in their originally classified folds. To classify a query, TM-scores are calculated between the query and all domains in a fold. The maximum TM-score observed represents the query-fold similarity, and is referred to as query-fold TM_max_-score. The query is assigned to the fold with the highest query-fold TM_max_-score. We repeat this entire process 30 times to estimate the frequency of inconsistency between the TM_max_-based classification and the CATH classification.

**Figure 1:**
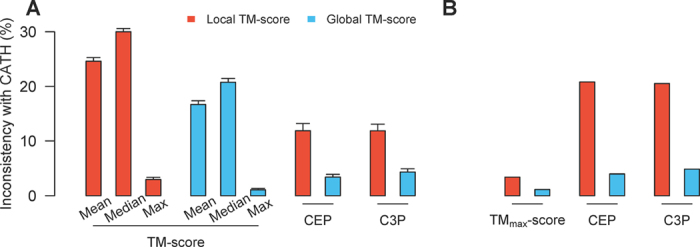
Fractions of fold-level domain structure classifications by various TM-scores, CEP, and C3P that are inconsistent with the CATH classification for (**A**) 30 sets of 10% randomly chosen domains from CATH v3.5.0 and (**B**) 8280 newly added domains in CATH v4.0.0 since v3.5.0. Error bars show one standard deviation.

Our local TM_max_-score-based classification is inconsistent with the current CATH fold classification for an average of 2.9% of queries (Fig. 1). This value decreases to 1.1% under the global TM_max_-score-based classification (Fig. 1). We also tried using either the mean or median TM-score instead of TM_max_-score to define domain-fold similarity, but the frequency of inconsistency rises to 17–30% (Fig. 1). These results indicate that the CATH fold classification is primarily based on the information contained in TM_max_-scores, especially in terms of the global structural similarity. Thus, TM_max_-score-based fold classification, which can be fully automated, may be used as a proxy for CATH classification.

### Within-fold structure heterogeneity varies among folds

**Figure 2:**
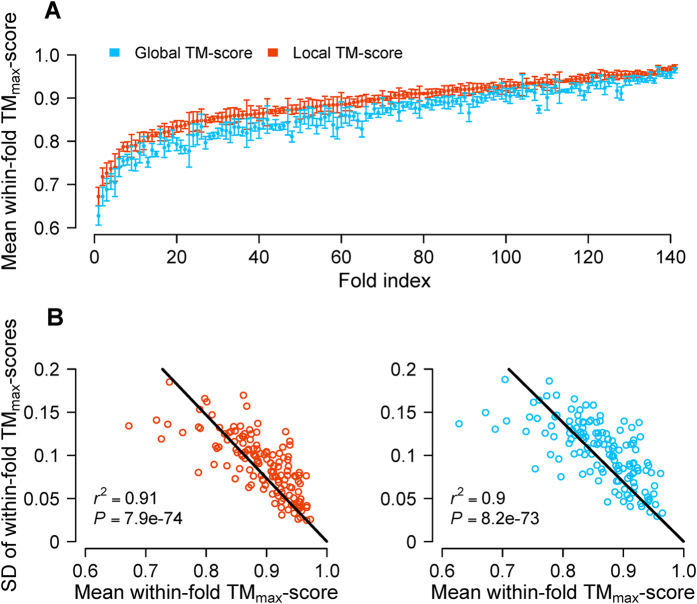
Within-fold structural heterogeneities of the 141 large folds in CATH version 3.5.0. (**A**) Mean within-fold TM_max_-score of each fold. A whisker indicates the standard deviation (SD) of the within-fold TM_max_-scores. (**B**) Correlation between the mean and SD of within-fold TM_max_-scores across folds.

Different folds in the current CATH classification may have different levels of structure heterogeneity. To measure structure heterogeneity within a fold, we first calculated the (local or global) TM_max_-score for each domain in the fold, which is defined by the highest TM-score between the focal domain and all other domains in the fold. We then calculated the mean and standard deviation of the TM_max_-scores of all domains in the fold. The mean within-fold TM_max_-score is a measure of structure homogeneity within a fold, because the higher the mean within-fold TM_max_-score, the higher the structure homogeneity within the fold. Our analysis reveals that some folds are highly homogenous with the mean within-fold TM_max_-score approaching 1, whereas some other folds are highly heterogeneous with the mean within-fold TM_max_-score as low as 0.6-0.7 (Fig. 2A). Furthermore, the standard deviation of within-fold TM_max_-scores also varies greatly among folds and a very strong negative correlation exists between the mean and standard deviation of within-fold TM_max_-scores (Fig. 2B). This latter observation indicates that, when a fold has a low mean TM_max_-score, it is typically because some of the within-fold TM_max_-scores are very low rather than all within-fold TM_max_-scores are low.

### A likelihood method for fold classification considering within-fold structure heterogeneity

**Figure 3:**
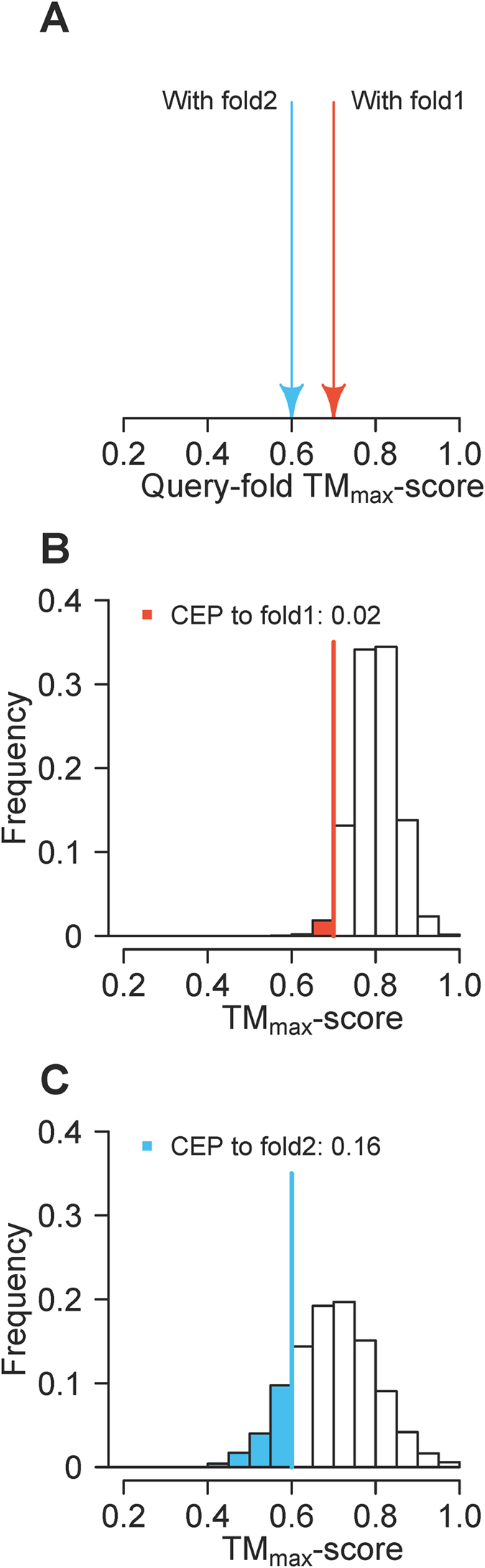
A hypothetical example contrasting fold classifications by TM_max_-score and CEP. (**A**) query-fold TM_max_-scores of a query to two folds. (**B**) Frequency distribution of within-fold1 TM_max_-scores. (**C**) Frequency distribution of within-fold2 TM_max_-scores. In (**B,C**), CEP is the area left to the vertical line under the distribution.

How well a query fits a fold should not only be determined by the query-fold TM_max_-score, but also the distribution of within-fold TM_max_-scores; folds with wider distributions of within-fold TM_max_-scores are more accommodating to a query than those with narrower distributions. The likelihood that a query belongs to a particular fold can be measured by the fraction of within-fold TM_max_-scores equal to or smaller than the query-fold TM_max_-score. We refer to this fraction as the cumulative empirical probability (CEP). Note that CEP is a measure of the fit of a query-fold TM_max_-score to the TM_max_-scores of all members already classified to the fold. CEP is not the posterior probability that a query belongs to a fold, and the sum of CEPs for all folds is not necessarily 1. Figure 3 shows a hypothetical example where CEP classifies a query into fold2 despite that the query-fold2 TM_max_-score is lower than the query-fold1 TM_max_-score (Fig. 3A). This occurs because the fraction of within-fold TM_max_-scores that are equal to or smaller than the corresponding query-fold TM_max_-score is smaller for fold1 (Fig. 3B) than for fold2 (Fig. 3C). Note, however, that classifications by CEP and TM_max_-score would always be consistent if the fold space is completely discrete, because then the TM_max_-scores of a query with fold1 and fold2 would be extremely different.

Estimating CEP requires the information on the empirical distribution of within-fold TM_max_-scores. When the number of domains in a fold is not very large, CEP estimates may be inaccurate. For example, when the query-fold TM_max_-score is lower than all observed within-fold TM_max_-scores, one assigns CEP = 0, although the true CEP must be >0. To minimize this problem, we can fit the observed within-fold TM_max_-scores (*x*) by a Gaussian mixture model (GMM) and then estimate CEP using the fitted continuous distribution (see Materials and Methods). The use of GMM is inspired by the fact that (i) the distribution of within-fold TM_max_-scores usually has multiple modes presumably due to the existence of multiple superfamilies in the fold and (ii) that GMM is highly flexible and fits almost any distribution. The parameters of the GMM are inferred under the Bayesian framework with model settings proposed by Richardson and Green^39^. With the posterior distributions of the parameters, the posterior predictive distribution of TM_max_-scores 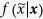 is estimated using a Monte Carlo method, where 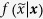 denotes the probability density of a potentially observed TM_max_-score 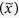 given the observed TM_max_-scores (*x*). CEP is then determined using 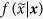 as if the potentially observed TM_max_-scores are actually observed. We refer to this CEP estimate as the **c**umulative **p**osterior **p**redictive **p**robability (C3P).

### Domain classification using CEP and C 3P with local TM_max_-scores

**Figure 4:**
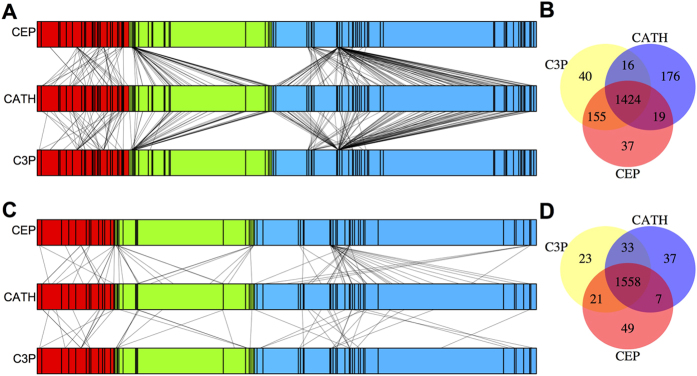
CEP and C3P classifications of 10% of randomly picked domains from large folds in CATH. (**A**) Reclassifications by local TM_max_-score-based CEP and C3P. A vertical bar corresponds to a fold, and its width is proportional to the number of domains in the fold. Domains within a fold are sorted by length ascendingly. The red, green, and blue colors represent folds from *α, β* and *αβ* classes in CATH, respectively. A line linking vertical bars of two horizontal bars connects the same domain that is classified into different folds by the two different methods, although a vertical bar with the same width of a line denotes 20 domains. This discrepancy is introduced for clearer visualization. (**B**) Venn diagram of classifications by CATH and local TM_max_-score-based CEP and C3P. (**C**) Reclassifications by global TM_max_-score-based CEP and C3P. (**D**) Venn diagram of classifications by CATH and global TM_max_-score-based CEP and C3P. In (**A**,**C**), results from the first of the 30 sets of queries are presented. In (**B**,**D**), average results from the 30 sets of queries are presented.

**Figure 5:**
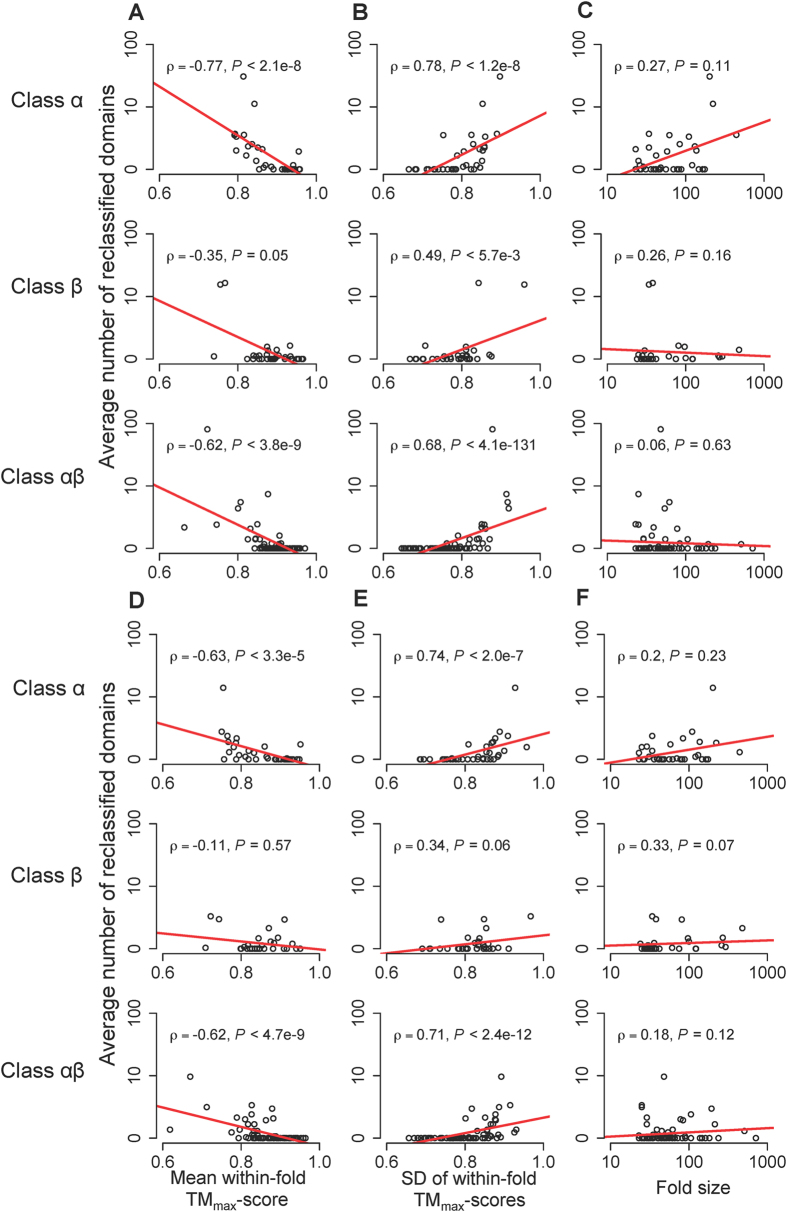
Rank correlations between various properties of a fold and the number of domains reclassified into the fold by (**A–C**) local TM_max_-score-based CEP and (**D–F**) global TM_max_-score-based CEP. The lines show linear regressions. ρ, Spearman’s rank correlation coefficient.

In this section, we use CEP and C3P with local TM_max_-scores for classification. This treatment is consistent with the focus on substructure similarity between domains in the study of fold space continuity. For the same 30 random sets of queries previously used, the CEP classification differs from the CATH classification in 12% of cases on average (Fig. 1). We refer to the query domains that have different classifications by CEP and CATH as reclassified domains. The majority of these domains are attracted to a small number of folds in CEP classification (Fig. 4A). These folds tend to have large structure heterogeneities (i.e., with low averages and high standard deviations of within-fold TM_max_-scores). In fact, the structure heterogeneity of a fold and the number of reclassified domains attracted to the fold are significantly correlated (Fig. 5A,B). By contrast, there is no significant correlation between the number of reclassified domains attracted to a fold and the fold size (Fig. 5C).

On average, C3P classification differs from the CATH classification in 12% of cases (Fig. 1), and the reclassified queries by C3P are also attracted to a small number of folds (Fig. 4A). As expected, the general patterns of C3P reclassifications are similar to what was observed in CEP reclassifications (Fig. S1A–C). Averaged over the 30 query sets, 97% of the queries are classified consistently by CEP and C3P (Fig. 4B). Moreover, 81% of the reclassifications by CEP are reclassified the same way by C3P, and 79% of the reclassifications by C3P are reclassified the same way by CEP (Fig. 4B).

### Domain classification using CEP and C3P with global TM_max_-scores

Let us now use global TM_max_-scores in CEP and C3P classifications. For the 30 sets of queries, CEP and C3P classifications differ from CATH classification for 3.4% and 4.3% of cases (Fig. 1), suggesting that the impact of fold space continuity on fold classification is substantially reduced if global structure similarity is considered. Similar to what was observed in the previous section, the number of domains reclassified into a fold correlates with measures of the fold’s structure heterogeneity (Fig. 5D,E; Fig. S1D,E), but is uncorrelated with the number of domains in the fold (Fig. 5F; Fig. S1F). CEP and C3P classifications are consistent with each other for 97% of cases (Fig. 4D). Thirty percent of the reclassifications by CEP are reclassified the same way by C3P, while 48% of the reclassifications by C3P are reclassified the same way by CEP (Fig. 4D).

### Why the reclassification rate is lower under global than under local TM_max_-scores

**Figure 6:**
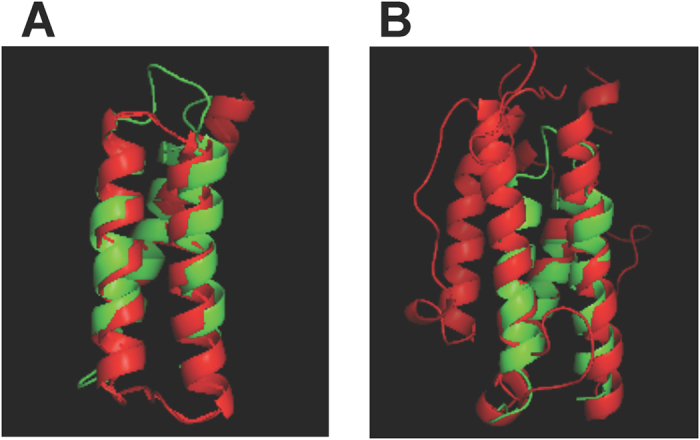
Structure alignments of a CATH domain based on local TM_max_-scores. The green structure shows the query domain (CATH id = 1lq7A00). The query is reclassified into fold 1.20.120 from 1.20.1270 by CEP based on local TM_max_-scores. (**A**) Structure alignment with 2oo2A00 (red) of fold 1.20.1270 (query-fold TM_max_-score = 0.73). (**B**) Structure alignment with 2qe9A01 (red) of fold 1.20.120 (query-fold TM_max_-score = 0.73).

Considering structure space continuity leads to reclassifications of only ~4% of domains under global TM_max_-scores, compared with ~12% under local TM_max_-scores. This increased reclassification rate under local TM_max_-scores is potentially due to the Russian doll effect^3^, which refers to the phenomenon that one domain resembles a substructure of another domain across folds. For example, domain 1lq7A00 (CATH Id) is classified into fold 1.20.1270 by CATH. However, this query domain has a local TM_max_-score of 0.73 with both folds 1.20.1270 (Fig. 6A) and 1.20.120 (Fig. 6B), because the query is highly similar to part of the domain structures in fold 1.20.120 (Fig. 6B). As a result, the query is classified by both CEP and C3P into fold 1.20.120, which is more heterogeneous in structure (mean within-fold TM_max_-score = 0.82) than fold 1.20.1270 (mean within-fold TM_max_-score = 0.87). By contrast, the query has a much smaller global TM_max_-score with fold 1.20.120 (0.48) than fold 1.20.1270 (0.70), and thus is classified into fold 1.20.1270 by CEP/C3P, consistently with the CATH classification.

### Classification of newly solved domain structures in CATH by CEP and C3P

The query domains used in previous sections were randomly chosen from the 17,043 representative domains in CATH v3.5.0. These queries are unbiased samples and their reclassification results by CEP and C3P represent the overall impact of structure space continuity on fold classification. However, if we need to classify a newly solved domain structure into the current CATH fold hierarchy, how big of an impact would the use of CEP or C3P have? To address this question, we took the 17,043 representative domains from the 141 large folds in CATH v3.5.0 (available from Sept., 2011) as the initial classification. In CATH v4.0.0 (available from March 2013), these large folds contain 8,280 representative domains that did not exist in CATH v3.5.0. We now use these 8,280 newly added domains as queries. When the local TM_max_-score is used, CEP (or C3P) classifications differ from CATH classifications for 20.8% (or 20.5%) of these 8,280 domains (Fig. 1B). When the global TM-score is used, CEP (or C3P) classifications differ from CATH classifications for 4.0% (or 4.8%) of these domains (Fig. 1B). These values are higher than the corresponding numbers for the 30 sets of randomly picked domains. This is potentially because folds were initially defined by some of the 17,043 domains, whereas a large fraction of the newly solved 8,280 domains may exist in uncharted regions between the initially defined folds. Consequently, such domains were assigned as boundary members of various folds by CATH, and thus tend to be reclassified by CEP/C3P.

### Classification of domains in the SCOP database

**Figure 7:**
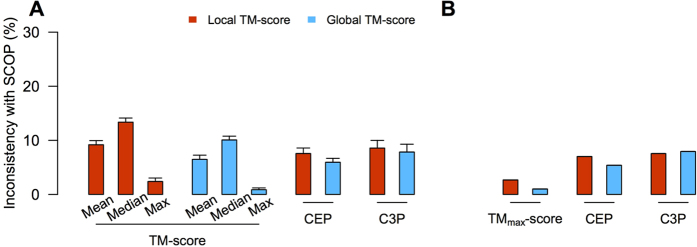
Fractions of fold-level domain structure classifications by various TM-scores, CEP, and C3P that are inconsistent with the SCOP classification for (**A**) 30 sets of 10% randomly chosen domains from CATH v3.5.0 and (**B**) 7050 newly added domains in SCOP v1.75 since v1.73. Error bars show one standard deviation.

We next examined the fold classification in SCOP, another widely used protein classification system. Using the same criteria as used for CATH, we generated 30 sets of 606 representative queries from 89 large folds in SCOP version 1.73. Local TM_max_-score-based fold classification is largely consistent with the SCOP classification, with only 2.4% of inconsistent cases (Fig. 7). This number decreases further to 0.9% under global TM_max_-score-based classification. The frequency of inconsistent classification is much greater when the query-fold similarity is measured by either the mean or median TM-score instead of TM_max_-score (Fig. 7). These results indicate that, similar to CATH, SCOP fold classification can be automated using query-fold TM_max_-scores.

For the same 30 random sets of queries, the local TM_max_-score-based and global TM_max_-score-based CEP classifications differ from the SCOP classification for an average of 7.6% and 5.9% of queries, respectively (Fig. 7). These numbers become 8.6% and 7.8%, respectively, for local and global TM_max_-score-based C3P classifications, respectively (Fig. 7).

By comparing SCOP versions 1.73 (available from Nov. 2007) and 1.75 (available from June 2009 and the most updated version), we found that 7050 representative domains were added into the 89 large folds in version 1.75 since version 1.73. These most recent additions to SCOP were subject to CEP and C3P classifications. The local and global TM_max_-score-based CEP classifications of these domains are inconsistent with the SCOP classification for 7.0% and 5.4% of the cases, respectively. These numbers become 7.5% and 8.0%, respectively, under C3P.

**Figure 8:**
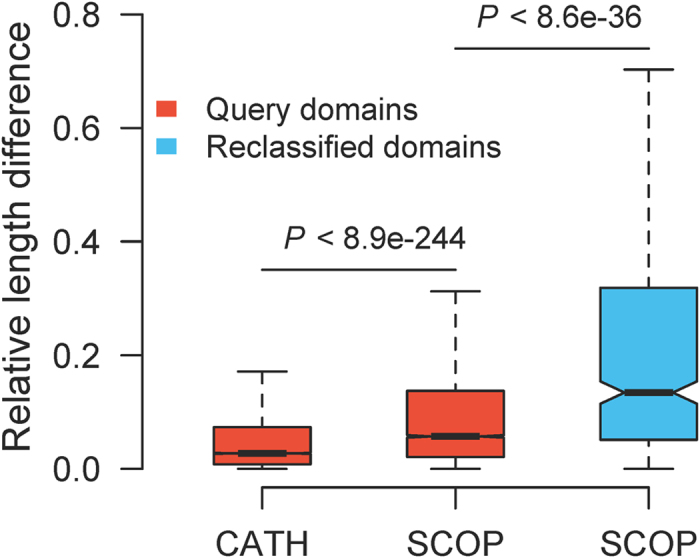
Relative length difference between domains within folds. The relative length difference is defined as the absolute length difference between a query from a fold and its best-matched domain (i.e., with the highest TM-score) in the fold, divided by the length of the shorter of the two. Domains included in the CATH bar are 16,173 nonredundant domains from the 30 sets of random queries in CATH v3.5.0. Domains included in the red SCOP bar are 6,134 nonredundant domains from the 30 sets of random queries in SCOP v1.73. Domains included in the blue SCOP bar are 456 queries reclassified by global TM_max_-score-based CEP. In this bar plot, the notch indicates the median and the bar corresponds to the interquartile range (IQR), covering from the first quartile to the third quartile of the sample. The two whiskers of the bar show the minimum value not smaller than the 1^st^ quartile minus 1.5 times IQR and the maximum value not greater than the 3^rd^ quartile plus 1.5 times IQR, respectively. *P* values are from Mann-Whitney *U* tests.

Reclassifications are rarer for SCOP than for CATH except under global TM_max_-score-based CEP and C3P (Figs 1 and 7). The SCOP data used here comprise 89 large folds and 65% of the total 9,964 representative domains in v1.73, whereas the CATH data consist of 141 large folds and 82% of the total 21,309 representative domains in v3.5.0. The sparser SCOP data than CATH data may render the classification more straightforward for the former than the latter. Intriguingly, however, global TM_max_-score-based CEP and C3P classifications are less consistent with SCOP than CATH classifications. To identify the underlying reason, we focus on the CEP classifications of the 6,134 non-redundant queries in the 30 SCOP sets. Each query has an original fold assigned by SCOP. The domain used to calculate query-original fold TM_max_-score is referred to as the partner domain. The relative length difference between the query and the partner domain is defined by the absolute value of their length difference divided by the shorter length. We found the relative length difference significantly greater for SCOP than CATH queries (Fig. 8). Because length difference reduces global TM_max_-scores, query-original fold global TM_max_-scores are reduced more drastically for SCOP than CATH queries, resulting in more reclassifications for the former than the latter. Indeed, reclassified SCOP queries tend to have larger relative length differences with their partner domains than average SCOP queries (Fig. 8).

### Domain classification using CEP with HHsuite

HHsuite package^40^,^41^ is widely used to predict protein fold from sequences^42^. Therefore, it is important to examine its sensitivity to fold space continuity. To this end, we used HHsuite to classify the new CATH domains into the existing folds. HHsuite first generates a hidden Markov model (HMM) for each protein sequence, and then aligns HMMs with two alternative dynamic programing algorithms that are derived respectively from Smith-Waterman^43^ and Needleman-Wunsch^44^ algorithms. For each alignment, three quantities, Probability, *P*-value, and Raw score, may be used for fold classification. The Raw score measures the sequence similarity of two proteins. The *P*-value is the probability that an alignment of non-homologous proteins will score at least the observed Raw score. The Probability measures the probability that the aligned proteins are homologous, by considering both the Raw score and the similarity of the predicted secondary structures of the proteins. For the newly solved CATH domains, the classifications based on the six quantities (i.e., two alignments each with three scores) differ from the CATH classification in only 2~3% of cases (Fig. S2). These numbers increase to 10–14% when CEP is used with the six quantities (Fig. S2). Thus, fold space continuity affects HHsuite-based fold classification.

## Discussion

In this work, we first showed that the fold classification in CATH is highly similar to the classification by the query-fold TM_max_-score, especially the global TM_max_-score. Considering fold space continuity, we developed the CEP and C3P methods to classify domain structures into existing folds using both query-fold TM_max_-scores and within-fold TM_max_-scores. When substructure similarity is concerned, fold classification is substantially influenced by the structure space continuity (12–20.8%). This conclusion also holds when the CEP uses HHsuite scores instead of TM-scores. Therefore, it is generally not legitimate to ignore this continuity under such scenarios. By contrast, when overall structure similarity is concerned, fold space continuity has only minor impacts on fold classification (3.4–4.8%) and thus may be ignored. The above results may have important implications for protein function prediction using structure similarity, because similar functions may require substructure similarity but not necessary global structure similarity. In other words, considering fold structure continuity may improve protein function prediction.

Under the global TM_max_-score, considering fold space continuity leads to the reclassification of 5 -8% SCOP domains, compared to 3 ~ 4% of CATH domains. This increased reclassification rate for SCOP domains is potentially due to the stronger Russian doll effect within SCOP folds than CATH folds, indicated by the higher within-fold length heterogeneity in SCOP than CATH (Fig. 8). This renders the global query-original fold TM_max_-score lower for SCOP queries than CATH queries, prompting more reclassifications for the former than the latter. The higher length heterogeneity in SCOP than in CATH may be due to different protocols and tools used in their classifications.

We found that the classification using global query-fold TM_max_-scores is inconsistent with CATH classification for only 1% of cases, confirming that CATH classifies a new domain based on its best match to existing members of various folds. It is clear that this way of classification will result in the problem that some domains of the same fold are less similar to one another than to domains from other folds, which is observed in CATH^33^. For example, domains A and B with low similarity to each other may be classified into the same fold because of their respective high similarities to some existing members in the fold. Non-transitive domain pairs such as A and B were observed previously^3^,^13^, but its prevalence and impact on classification in CATH were unclear. Unlike the query-fold TM_max_-score, the query-fold TM_mean_-score is influenced by the non-transitive domain pairs within a fold. The classification using TM_mean_-score differs from CATH for ~20% of cases. The substantial rise in inconsistency suggests that non-transitive domain pairs within a fold are quite common in CATH.

As mentioned, two types of methods have been developed to classify domain structures into folds. The first type is similar to TM_max_-score and does not consider the impact of fold space continuity^20^,^21^,^22^,^23^,^24^,^25^,^26^,^27^. The second type is based on the machine learning approach^28^,^29^,^30^,^31^ and is trained with within-fold domain pairs from multiple folds. However, pooling domain pairs from many folds for training ignores the among-fold variation in within-fold structure heterogeneity, by which fold space continuity affects current classifications. One might think that training the classifiers with individual folds can solve this problem, but it is infeasible because of small samples of most folds that cause overfitting of the classifiers with large numbers of parameters. Interestingly, although the classifiers neglect fold space continuity, their classification results are still substantially (~20%) different from current fold classifications. This is likely due to the uses of non-transitive domain pairs to train the classifiers. Overall, CEP and C3P are unique in that they explicitly consider structure space continuity in fold classification. In addition, C3P is based on Bayesian hierarchical models that alleviate overfitting.

In summary, we have developed CEP and C3P to estimate the impact of fold space continuity on current fold classifications. The inconsistencies between CEP/C3P and current hierarchical classifications in CATH and SCOP demonstrate a substantial impact of structure continuity on fold classification when local structure similarity is considered. By contrast, for questions that concern global structure similarities, the current fold classifications are largely valid. In our analysis, we classified query domains into existing folds, which were established without considering fold space continuity. In the future, it would be interesting to develop model-based clustering of all domains where the number of folds and memberships in each fold are both probabilistic.

## Methods

### Protein structure similarity score

TM-score defined below is used to assess the structural similarity between two protein structures. 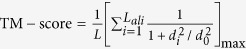, where *L* is the length of the shorter protein (local TM-score) or mean length of the two proteins being compared (global TM-score), *L*_*ali*_ is the number of equivalent residues in the two proteins, *d*_*i*_ is the distance of the *i* th pair of the equivalent residues between the two superposed structures, 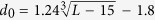 is used to normalize the TM-score so that the average magnitude of the TM-score for random protein pairs is independent of the size of the proteins, and “max” indicates the highest value among all possible superpositions. TM-score ranges in (0, 1] with a higher value indicating a higher similarity. TM-scores between two domains are calculated using the TM-align software^34^.

### Fold recognition using HHsuite

The HHsuite package was used to measure structure similarity between two protein sequences. For each protein alignment, Probability, *P*-value, and Raw score calculated by HHsuite were respectively used to measure structure similarity. HHsuite provides two alternative algorithms of dynamic programming, derived from Smith-Waterman and Needleman-Wunsch algorithms, respectively. We considered each of these two algorithms, coupled with default values for all other parameters except that realignment was not allowed due to its high computational cost.

### Initial classifications and query domains

From CATH v4.0.0, we collected 23,682 representative single domains with mutual sequence identities ≤60% and lengths ≥40 residues using CD-Hit^45^. Among them, 21,309 representative domains existed in an older version of CATH (v3.5.0). These 21,309 domains are from 1,158 folds in CATH v3.5.0. A total of 141 of these folds each have at least 25 domains. From each of these 141 large folds, we randomly sampled 10% of domains; these 1,635 queries sampled were subjected to classification by other methods. The remaining 15,408 domains in the 141 folds constitute the initial fold classification. This procedure was repeated 30 times, generating 30 random sets of queries. To examine the CATH classifications of newly solved domain structures, representative domains of the 141 large folds in CATH v3.5.0 were used as the initial classification, whereas the 8,280 domains newly added to the 141 folds in CATH v4.0.0 were queries. With the same criterion, 6,476 representative domains in 89 large folds were collected from SCOP v1.73. Using only the large folds, 30 sets of query domains were randomly picked. Each of the sets contained 606 queries, and the other 5,870 domains in large folds were treated as the initial classifications. A total of 7,050 domains newly added to the 89 folds in SCOP v1.74 since v.1.73 were identified as newly solved queries.

### Gaussian mixture model and posterior predictive distribution

The observed within-fold TM_max_-scores for a fold are denoted as ***x***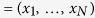, which are assumed to have been independently drawn from a mixture of *k* Gaussian components. *N* is the number of representative domains in the fold. The probability of observing an *x* is


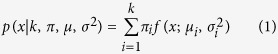


where *μ*_*i*_, 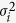, and *π*_*i*_ are the mean, variance, and mixture proportion of component *i*. Latent allocation data are referred to as 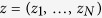, in which *z*_*i*_ specifies the mixture component to the observation 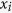. Here, *z*_*i*_’s are independently and identically distributed samples from the following probability mass function (PMF).


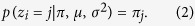


Conditional on the allocation value *z*_*i*_, the observed *x*_*i*_ is a random number from the following Gaussian probability density function.


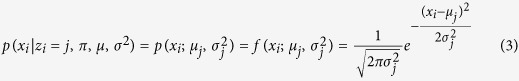


We assume the following priors of the parameters:


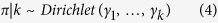



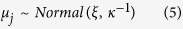



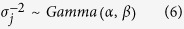



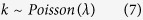


We set *γ*_*i*_ = 1, 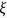 = median of observed TM_max_-scores of a fold, and 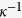 = 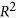,where *R* is the difference between the maximum and minimum of ***x***. These parameters make the prior distributions of *π* and *μ*_*j*_ rather flat. We set 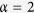 and 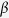
~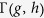, which is a gamma distribution with the shape parameter *g* = 0.2 and rate parameter *h* equal to 10/*R*^2^, to express the belief that the 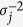
s are similar. At last, *k* follows a Poisson distribution with parameter *λ*=1. All the settings together render the priors weakly informative and thus allow observed TM_max_-scores to dominate the parameter inference. The joint prior probability can be written as


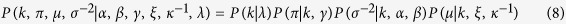


and therefore the joint posterior probability is


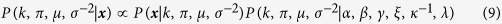


Let 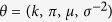 and 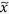 denote unobserved TM_max_-scores of the fold. The posterior predictive distribution is


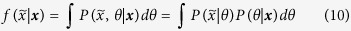


which is the probability density function (PDF) of potential query-fold TM_max_-scores (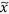) given the observed within-fold TM_max_-scores (*x*).

Due to the lack of a closed form, 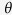
's are sampled from the posterior distribution 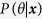 using reversible-jump Markov chain Monte Carlo implemented in the R package of miscF (https://CRAN.R-project.org/package=miscF). The simulation had 30,000 iterations, 5000 burn-in steps, and a thinning parameter of 5. Initial values unspecified previously are assigned automatically by the miscF package. Conditional on each *θ*, 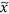 is sampled from the Gaussian mixture 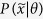. This model was used to develop the C3P method for fold classifications.

### CEP/C3P package and data availability

Both CEP and C3P methods are implemented in the C3P package, which can be obtained at http://www.umich.edu/~zhanglab/download.htm. All CEP and C3P classifications analyzed in this work are accessible at the same site.

## Additional Information

**How to cite this article**: Xu, J. and Zhang, J. Impact of structure space continuity on protein fold classification. *Sci. Rep.* **6**, 23263; doi: 10.1038/srep23263 (2016).

## Supplementary Material

Supplementary Information
