# Supplementary material for: Impact of structure space continuity on protein fold classification
Source: Sci Rep. 2016 Mar 23;6:23263. doi: 10.1038/srep23263 (PMC4804218; doi:10.1038/srep23263)
Supplement: Supplementary Information [file srep23263-s1.pdf]

Supplementary Materials for

Impact of structure space continuity on protein fold classification

Jinrui Xu and Jianzhi Zhang

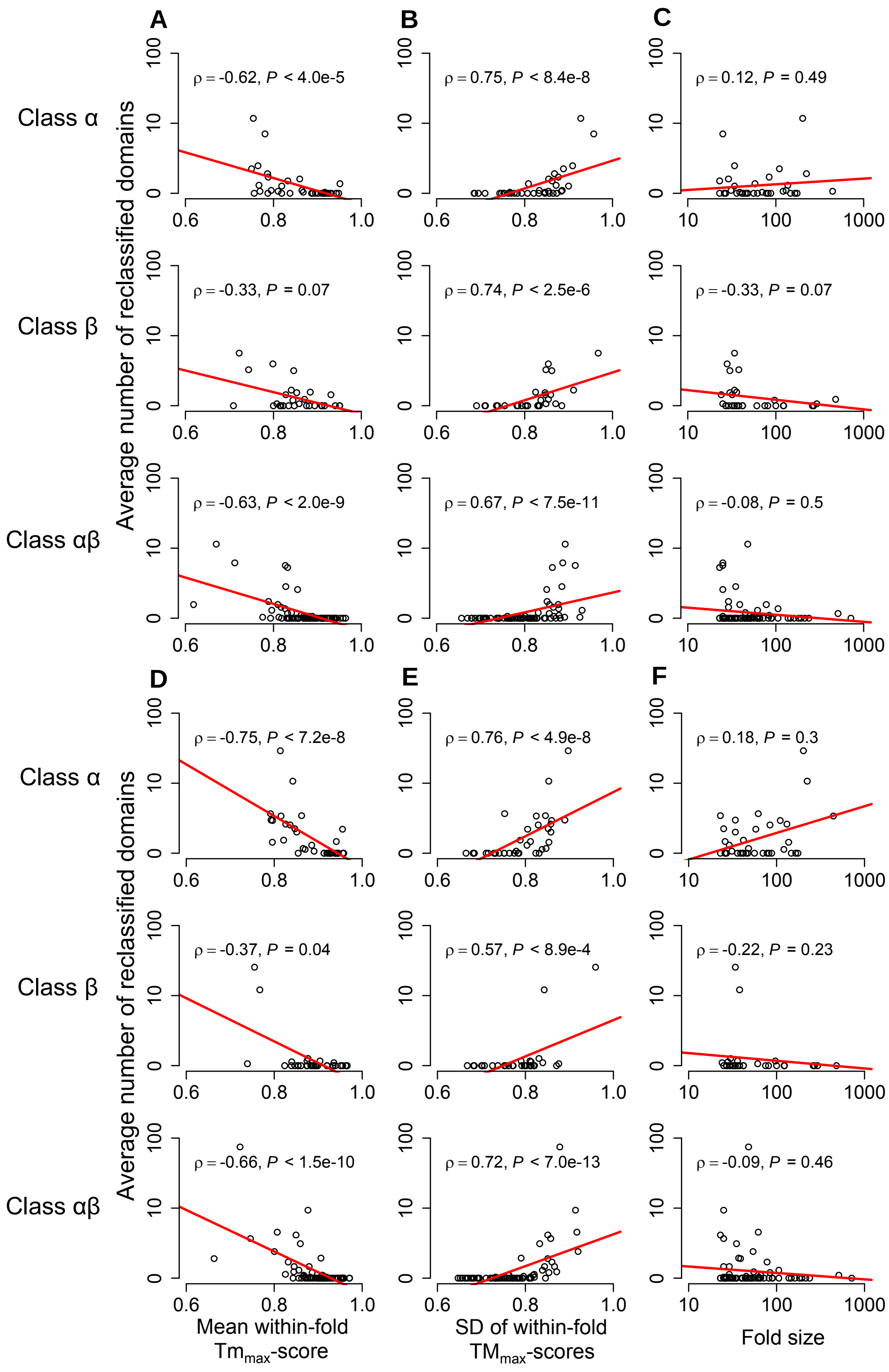

Fig. S1. Rank correlations between various properties of a fold and the number of domains reclassified into the fold by (**A-C**) global TM_{max}-score-based C3P and (**D-F**) local TM_{max}-score-based C3P. The lines show linear regressions. ρ, Spearman's rank correlation coefficient.

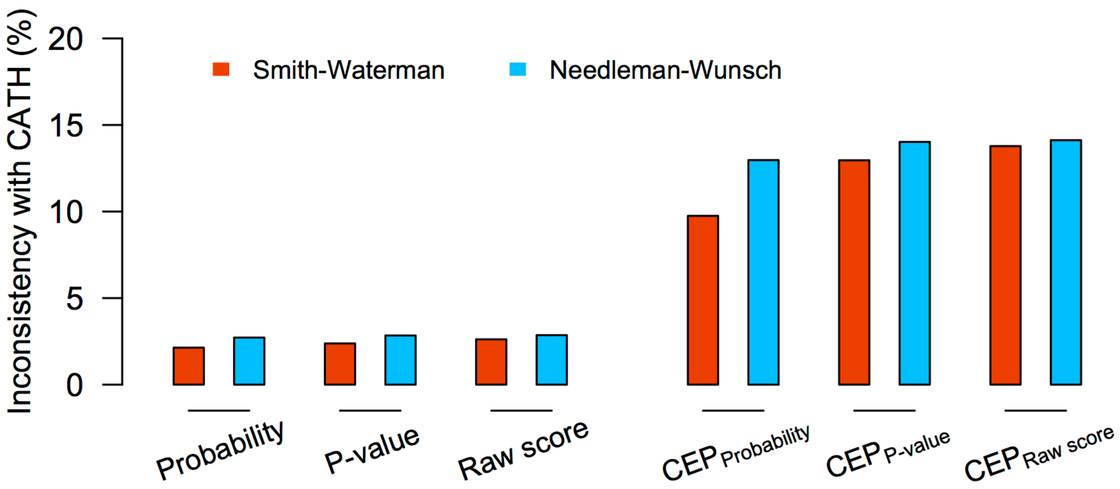

Fig. S2. Fractions of fold-level domain structure classifications by the similarity scores of HHsuite and the CEPs based on these similarity scores that are inconsistent with the CATH classification for the 8280 newly added domains in CATH v4.0.0 since v3.5.0.
